# Consequences of Interaction of Functional, Somatic, Mental and Social Problems in Community-Dwelling Older People

**DOI:** 10.1371/journal.pone.0121013

**Published:** 2015-04-21

**Authors:** Anne H. van Houwelingen, Wendy P. J. den Elzen, Saskia le Cessie, Jeanet W. Blom, Jacobijn Gussekloo

**Affiliations:** 1 Department of Public Health and Primary Care, Leiden University Medical Center, Leiden, the Netherlands; 2 Department of Clinical Epidemiology, Leiden University Medical Center, Leiden, the Netherlands; 3 Department of Medical Statistics and Bioinformatics, Leiden University Medical Center, Leiden, the Netherlands; Federal University of Rio de Janeiro, BRAZIL

## Abstract

This study explores the combination of four common health problems in older people and whether problems on four domains result in an additional effect on indicators of poor health. For this purpose, a total of 2681 participants (32% male, mean age 82 years) of the Integrated Systematic Care for Older People (ISCOPE) study were screened on the presence of health problems on four domains (functional, somatic, mental, social) with the postal ISCOPE questionnaire. Extensive interview data on health indicators were obtained at baseline and at 12-months follow-up, including disability (Groningen Activities Restriction Scale, GARS), cognitive function (Mini-Mental State Examination, MMSE), depressive symptoms (Geriatric Depression Scale-15, GDS), loneliness (loneliness scale of De Jong Gierveld), and health-related quality of life (EQ-5D). General practitioner (GP) contact time (min/year) was estimated via GP electronic medical records. Of the study population, 9% had no health problems according to the screening, 8% had problems on one domain, 27% on two, 38% on three and 18% on four domains. At baseline, the number of health domains with problems was associated with poorer scores on the GARS, the MMSE, the GDS-15, the loneliness scale, the EQ-5D and with more GP contact time (p <0.001). Problems on all four domains had an additional negative effect on these health indicators (all p_interaction_ <0.001). At follow-up, an increased number of domains with problems was associated with an increased decline in health indicators (all p<0.001) and with an additional negative effect on GP contact time of the presence of problems on all four domains (p_interaction_ <0.001). We conclude that combinations of functional, somatic, mental and social problems are associated with poor health indicators in community-dwelling older people. Since problems on four domains have an additional effect on health, individuals with combined functional, somatic, mental and social problems could benefit from integrated care.

**Trial registration:**

Netherlands Trial Register: NTR1946.

## Introduction

The prevalence of many diseases and conditions increases with age. As a result, older persons often have a variety of health problems including functional, somatic, mental and social problems. These problems have all been individually associated with poor health indicators including deteriorated functional status, decreased quality of life and increased healthcare use [1–16]. Older people often have a combination of multiple health problems. However, it is unclear if these problems interact and what the consequences are of the occurrence of a combination of health problems on functioning, wellbeing and healthcare use.

Therefore, this study investigates the consequences of problems on four health domains (functional, somatic, mental and social domain) on various health indicators, and whether the problems on four domains have an interactive effect on health indicators at baseline and at 12-month follow-up.

## Methods

### Ethical approval

The study was approved by the Medical Ethical Committee of Leiden University Medical Center in 2009.

### Study population

This study is embedded in the ISCOPE study (Integrated Systematic Care for Older PEople). ISCOPE is a cluster randomized controlled trial with randomization at general practice level. The overall aim of the ISCOPE study was to assess the efficacy of a simple structural monitoring system to detect deterioration in the functional, somatic, mental or social health of individuals aged ≥ 75 years, followed by a care plan for those people with a combination of problems on these domains. The study included 59 general practices (30 in the intervention group; 29 in the usual care group). After the general practitioners (GPs) excluded people with a life expectancy of ≤ 3 months, who were admitted to a nursing home, who were non-Dutch speaking, or who should be excluded for other relevant reasons (n = 590), 11,476 older persons received an invitation to participate in the study. The ISCOPE screening questionnaire (S1 File) and an informed consent form were sent with this invitation. In the intervention group, the GPs received feedback about the screening questionnaire, were trained in providing pro-active integrated care for older people, and made a care plan for a randomly selected sample of 10 older persons with a combination of functional, somatic, mental or social problems. In the usual care group, the GP did not receive feedback and provided usual care.

A sample of participants who returned the screening questionnaire was visited at home by a research nurse to collect data on sociodemographic characteristics and to administer additional questionnaires on health indicators; this sample included all participants with complex problems, a random 60% of the participants with problems on 2 domains, and a random 15% of the participants with problems on 0 or 1 domain. At the 12-month follow-up, these participants were revisited to obtain additional information.

All participants gave written informed consent for the study including the use of data from their medical records for additional analysis, following explanation of the study requirements and assurance of confidentiality and anonymity. For participants with severe cognitive impairment (as judged by the research nurses) written informed consent was obtained from a proxy. The Medical Ethical Committee of the Leiden University Medical Center approved the study and the informed consent procedure.

### Measurements

#### Identification of health domain problems at baseline

The ISCOPE screening questionnaire consists of 21 items which cover all 4 health domains (functional, somatic, mental and social domain; see Questionnaire S1) [17]. A participant was considered to have a problem on a domain, if a positive answer was given to two or more items on that domain.

#### Health indicators at baseline and follow-up

Disability in basic and instrumental activities of daily living (BADL and IADL) was measured with the Groningen Activities Restriction Scale (GARS) [18], a questionnaire that assesses disabilities in competence in nine BADL items and nine IADL items [19]. A sum score was calculated for the GARS, ranging from 18 (competent in all IADL and BADL activities) to 72 (unable to perform any activity without help).

Cognitive function was assessed with the Mini-Mental State Examination (MMSE) [20], with scores ranging from 0–30 (= optimal).

The presence of depressive symptoms was determined with the Geriatric Depression Scale-15 (GDS-15) [21]. The GDS-15 was restricted to people with a MMSE score of ≥ 19 since this instrument has proven unreliable for people with poor cognitive function [22].

Feelings of loneliness were measured with the Loneliness Scale of De Jong Gierveld [23], an 11-item scale designed for the elderly population. The maximum score was 11 points (severe loneliness). This loneliness scale was also restricted to people with a MMSE score of ≥ 19.

Health-related quality of life (HRQoL) at baseline and follow-up was evaluated with the EQ-5D [24], a standardized instrument for HRQoL.

To determine GP-contact time, data were extracted from the electronic patient records (EPR) of the participating GPs. The total number of contacts at baseline was defined as the sum of home visits and consultations in the year before the study was conducted. The total number of contacts at follow-up was defined as the sum of home visits and consultations in the year after the start of the ISCOPE trial. To estimate the GP contact time in minutes, a GP consultation was defined to be 10 min and a home visit was defined to be 30 min (including travelling time).

#### Additional characteristics

Data on sociodemographic characteristics, including sex, living situation, marital status, educational level and data on chronic diseases were obtained during the home interviews. Chronic diseases included diabetes, heart failure, malignancy, chronic obstructive pulmonary disease (COPD), incontinence, arthritis, osteoporosis, dizziness, lower urinary tract symptoms (LUTS), depression, anxiety, dementia, vision, deafness, fracture, stroke/transient ischemic attack (TIA) and myocardial infarction.

### Statistical analysis

Descriptive statistics on sociodemographic characteristics were calculated for the total study population and stratified for the number of domains with problems. P-values for a trend for differences within these groups were obtained with the linear-by-linear chi-squared test (dichotomous data) and with linear regression analysis, with the characteristic as the dependent variable and the number of domains with problems as the independent variable (continuous data).

Linear regression analysis was used to evaluate the association between the individual domains and the number of domains with problems and the health indicators at baseline and at follow-up. In each model the number of domains with problems, as well as sex and age, were included as independent variables. For the indicators at 12 months, the score on the health indicator at baseline was added as independent variable.

We examined whether having problems on 4 domains had more effect on the health indicators than expected (from the linear model) by including an interaction variable in the model that indicated whether problems on 4 domains existed (i.e. an additive interactive effect). This variable had the value of 1 when problems on four domains existed and 0 when there were no problems on four domains. The p-value for this additional variable was indicated as P_interaction_.

To investigate the relation between the number of domains with problems and dropout due to admission to a nursing home, severe (terminal) disease or death, a logistic regression analysis was performed with dropout (yes/no) as dependent variable. We also investigated whether poor cognitive function (MMSE <19) was related to dropout with the same model. The same independent variables were used as in the linear regression analysis.

A p-value <0.05 was considered to be statistically significant. Data were analyzed with the SPSS version 20.0 for Windows.

## Results

### Study population

**Fig 1:**
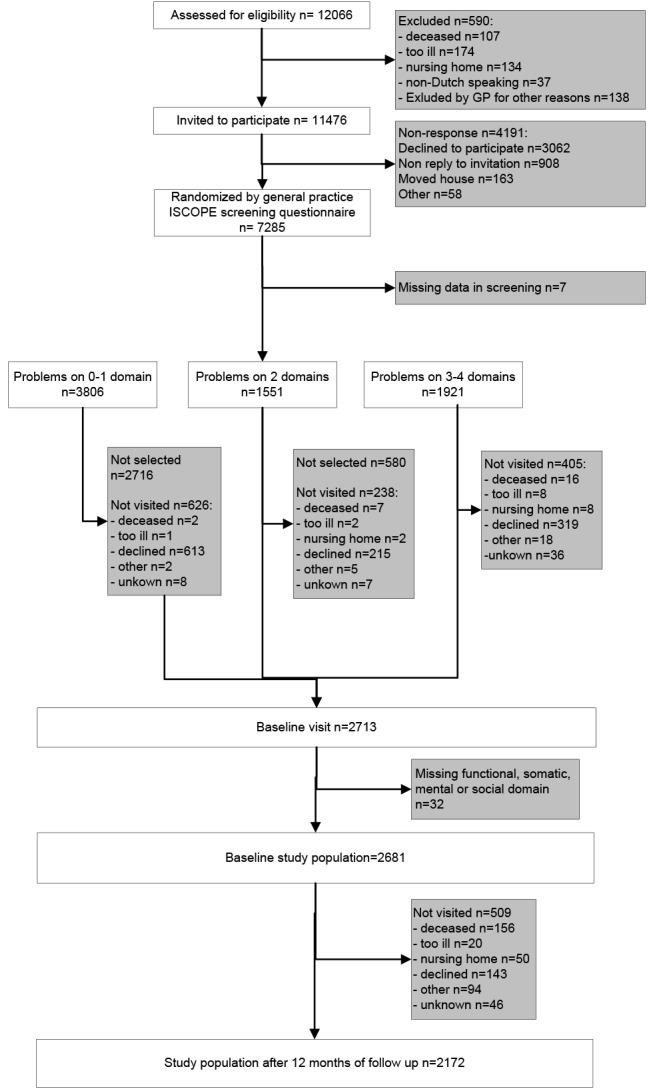
Flowchart of the study participants.

Of the 12,066 registered patients aged ≥75 years in the 59 general practices, 590 (4.9%) were excluded by the GP (Fig 1). Of the remaining 11,476 patients, 7285 (63%) participated and were randomized at the level of the general practice. Of these latter persons, a random sample of 2713 was visited at home to administer the outcome measurements. Because 32 participants had missing values on one of the domains of the ISCOPE screening questionnaire and were excluded, the final study population consisted of 2681 older people.

For the GP contact time, data were available for 1473 participants because some electronic systems did not allow data extraction. Of the 2681 participants, 105 (4%) had a MMSE-score below 19 points. These participants were excluded for the GDS-15 and the Loneliness Scale of De Jong Gierveld. Table 1 summarizes the baseline characteristics of the total study population and stratified for the number of domains with problems at baseline. The median age was 82 (Interquartile range [IQR] 78–86) years and 31.7% of the study population was male.

**Table 1:** Baseline characteristics of the total study population and stratified for the number of domains with problems.

	Total study population	Number of domains with problems					
		0	1	2	3	4	
	n = 2681	n = 243	n = 213	n = 720	n = 1027	n = 478	p-trend
Age in years	82 (78–86)	79 (76–83)	80 (77–84)	81 (78–81)	82 (79–86)	84 (80–88)	<0.001
Male sex	850 (31.7)	118 (48.6)	79 (37.1)	225 (31.3)	325 (31.6)	103 (21.5)	<0.001
Low income (state pension only)	404 (15.1)	24 (9.9)	21 (9.9)	104 (14.4)	160 (15.6)	95 (19.9)	<0.001
Low level of education (primary school only)	967 (36.1)	65 (26.7)	69 (32.4)	248 (34.4)	374 (36.4)	211 (44.1)	<0.001
Living alone	1461 (54.5)	98 (40.3)	108 (50.7)	398 (55.3)	612 (59.6)	245 (51.3)	0.001
Widowed	1424 (53.1)	76 (31.3)	95 (44.6)	359 (49.9)	591 (57.5)	303 (63.4)	<0.001
Care home	296 (11.0)	6 (2.5)	8 (3.8)	59 (8.2)	99 (9.6)	124 (25.9)	<0.001
Number of chronic diseases*	4 (3–6)	2 (1–4)	3 (2–4)	4 (3–5)	5 (3–6)	6 (4–7)	<0.001

Data are numbers (%) or medians (Inter quartile range).

p-trend values were obtained with linear-by-linear tests (categorical data) or linear regression analysis (continuous data).

* Including self-reported diabetes, heart failure, malignancy, COPD, incontinence, arthritis, osteoporosis, dizziness, LUTS, depression, anxiety, dementia, vision, deafness, fracture, stroke/TIA, myocardial infarction.

### Identification of problems at baseline

Of the study population, 243 participants (9%) had problems on no domain, 213 (8%) had problems on 1 domain, 720 had problems on 2 domains (27%), 1027 had problems on 3 domains (38%), and 478 participants had problems on all 4 domains (18%). The three combinations of problems with the highest prevalence were the combination of problems on the somatic, mental and social domain (n = 562, 21%), the combination of problems on all four domains (n = 478, 18%) and the combination of problems on the functional, somatic and mental domain (n = 341, 13%) (S1 Table). The age of the older persons increased across the increasing number of domains with problems, and the number of males decreased (Table 1; both p-trend <0.001).

A total of 1140 participants (43%) had problems on the functional domain, 2041 (76%) on the somatic domain, 1980 (74%) on the mental domain, and 1485 (55%) on the social domain. For all domains, participants with problems on any particular domain had poorer scores on health indicators (S2 Table), including higher GARS scores, higher GDS-15 scores and higher scores on the Loneliness scale, poorer EQ-5D scores and more GP contact time (all p < 0.001). Participants with problems on the functional, somatic or mental domain had lower MMSE scores (all p <0.001, S2 Table), while MMSE scores showed no difference between participants with and without problems on the social domain (p = 0.68, S2 Table).

### Associations at baseline

**Fig 2:**
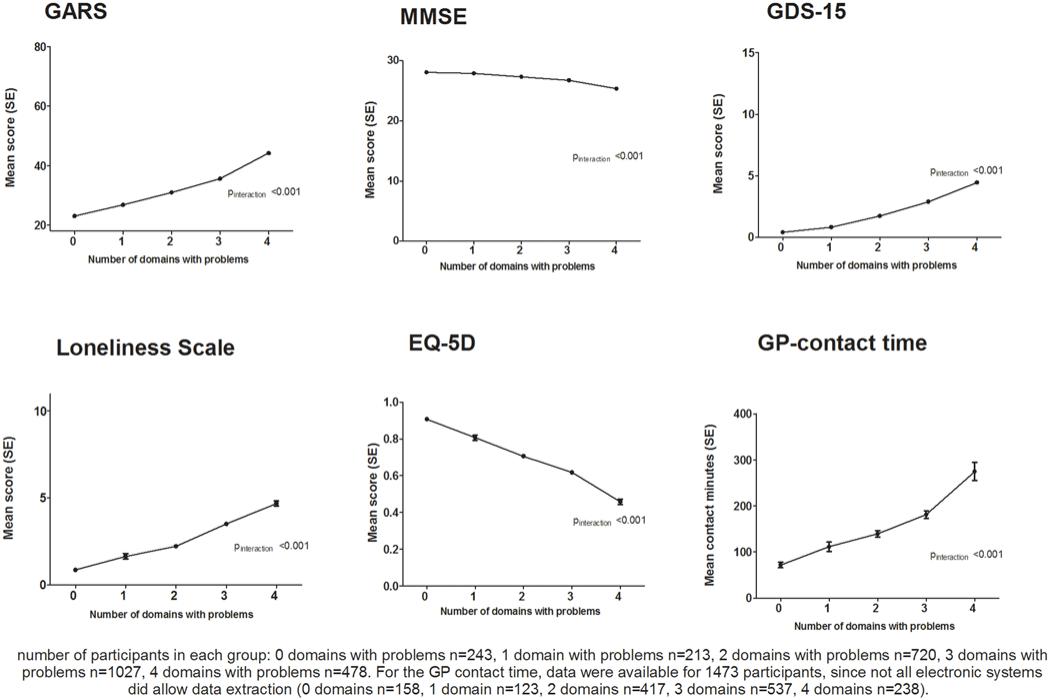
Association between the number of domains with problems at baseline and scores on the health indicators.

Fig 2 shows the association between the number of domains involved at baseline and scores on the health indicators at baseline. The number of domains with problems at baseline was associated with poor health indicators; per additional domain the GARS score was higher, the MMSE score was lower, the GDS-15 score was higher, the score on the Loneliness Scale of De Jong Gierveld was higher, the EQ-5D score was lower, and the GP contact time was higher (all p<0.001) (Table 2). Having four domains with problems had an additional negative effect on these indicators of poor health (all p<0.05) (Table 2).

**Table 2:** Association between the number of domains with problems on the health indicators at baseline (n = 2681)*.

	Effect of extra domain with problems at baseline		Additional effect of four domains with problems at baseline	
	β (95% CI)	p-value	β (95% CI)	p-value
GARS	3.74 (3.32;4.17)	<0.001	3.86 (2.60;5.13)	<0.001
MMSE	0.92 (0.82;1.03)	<0.001	0.78 (0.47;1.09)	<0.001
GDS-15	0.93 (0.81;1.04)	<0.001	0.40 (0.06;0.75)	0.023
Loneliness Scale of De Jong Gierveld	-0.09 (-0.11;-0.08)	<0.001	-0.06 (-0.09;-0.03)	<0.001
EQ5D	-0.40 (-0.54;0.25)	<0.001	-0.87 (-1.3;-0.44)	<0.001
GP contact time (min)‡	29 (19;40)	<0.001	49 (17;81)	<0.001

*linear regression analysis with health indicator at baseline as outcome and the number of domains with problems, as well as age and sex, as covariates.

^‡^ GP contact information was available for 1473 participants.

Abbreviations GARS = Groningen Activity Restriction Scale, GDS-15 = Geriatric Depression Scale, MMSE = Mini-Mental State Examination, EQ-5D = Euroqol-5D, GP = general practitioner.

### Effects at follow-up

At the 12-month follow-up, 509 participants were not visited because they were deceased (n = 156), too ill (n = 20), admitted to a nursing home (n = 50), declined further participation (n = 143) or for other (n = 94) or unknown reasons (n = 46), resulting in a sample of 2172 participants. For GP contact time, data were available for 1422 participants at 12 months follow up, because some electronic systems did not allow data extraction. Dropout was related to the number of domains with problems; per additional domain the odds to dropout from the study was odds ratio (OR)1.67 (95% CI 1.33–2.08; p <0.001) times higher. Having four domains with problems was not associated with an additional increased risk of dropout (OR 0.95 [95% CI 0.61–1.49], p_interaction_ = 0.82). The odds to dropout from the study for participants with impaired cognition (MMSE-score <19) was OR 2.54 (95% CI 1.68–3.84; p <0.001) times higher, compared to participants with MMSE score of 19 points and higher.

At the 12-month follow-up, problems on the functional domain at baseline were individually associated with poorer GARS-, MMSE-, GDS-15-, and EQ-5D- scores (linear regression analysis adjusted for the baseline score on the health indicator, age and sex, all p <0.001, S3 Table). Problems on the somatic domain and on the mental domain at baseline were individually associated with poorer scores on all these health indicators at follow up (all p <0.05, S3 Table). Problems on the social domain at baseline were associated with poorer GDS-15-, loneliness- and EQ-5D scores, and with more GP-contact time.

The number of domains with problems at baseline was associated with poorer scores on the GARS, GDS-15, the Loneliness Scale of De Jong Gierveld and the EQ-5D, and with more GP contact time, adjusted for the baseline score of the health indicator, as well as age and sex (Table 3). For MMSE, there was no significant association between the number of domains with problems and the score at 12-months follow-up (p = 0.096), but when participants had problems on all four domains, they showed an additional decrease in MMSE-score of -0.4 points (p = 0.057). For GP contact time at 12-months follow-up, there was an additional negative effect of having 4 domains with problems on GP contact time (52 min higher [95% CI 23; 81], P_interaction_<0.001).

**Table 3:** Effect of the number of domains with problems on health indicators at 12-month follow-up (n = 2172)*.

	effect of baseline score of health indicator		effect per domain with problems at baseline		additional effect of 4 domains with problems at baseline (interaction)	
	β (95% CI)	p-value	β (95% CI)	p-value	β (95% CI)	p-value
GARS	0.9 (0.9;0.9)	<0.001	0.7 (0.4;1.0)	<0.001	0.5 (-0.4;1.5)	0.271
MMSE	1.0 (0.9;1.0)	<0.001	-0.1 (-0.3;0.02)	0.096	-0.4 (-0.8;0.01)	0.057
GDS-15	0.6 (0.6;0.7)	<0.001	0.3 (0.2;0.4)	<0.001	0.2 (-0.1;0.5)	0.25
Loneliness Scale of De Jong Gierveld	0.6 (0.6;0.7)	<0.001	0.3 (0.2;0.4)	<0.001	-0.2 (-0.5;0.1)	0.28
EQ5D	0.4 (0.4;0.4)	<0.001	-0.06 (-0.07;-0.04)	<0.001	0.01 (-0.02;0.04)	0.69
GP-contact time (min)‡	0.7 (0.7;0.8)	<0.001	13 (3.5;22)	<0.001	52 (23;81)	<0.001

*linear regression analyses with health-indicator after 12 months as outcome and baseline score of health indicator, number of domains with problems, four domains with problems present, and age and sex as covariates.

Abbreviations GARS = Groningen Activity Restriction Scale, GDS-15 = Geriatric Depression Scale, MMSE = Mini-Mental State Examination, EQ-5D = Euroqol-5D, GP = general practitioner.

^‡^ GP contact information was available for 1422 participants at 12 month follow up.

## Discussion

In the present study, the number of problems on the health domains included in the ISCOPE screening questionnaire was associated with poor scores on health indicators, including disability, impaired cognition, depressive symptoms, feelings of loneliness, HRQoL and GP contact time, both at baseline and at follow-up.

Problems on 4 health domains had an interactive negative effect on disability, cognitive function, feelings of loneliness, HRQoL and GP contact time at baseline. Having problems on 4 domains was associated with an additional negative effect on GP contact time at the 12-month follow-up.

The results of the present study confirm the associations between the individual health domains and poor health indicators as reported previously [1–16]. However, to our knowledge, we are the first to show that having 4 domains with problems has additional negative consequences for older persons. It is known that multisystem physiological dysregulation has a nonlinear association with poor outcomes in older adults and that dysregulation in physiological systems increases with old age [25–27]. Moreover, paired combinations of chronic conditions have been found to have interactive effects on disability [28]. In addition, combinations of frailty with disability and multimorbidity have been proven to have interactive effects on death [29], quality of life and health care costs [30]. The present study shows that, apart from the physiological (somatic) domain and the functional domain, two other domains (mental and social) are also related to poor health and wellbeing in older individuals, and that the negative consequences increase nonlinearly when all 4 of these domains are affected. This nonlinearity might reflect the complexity that arises when several systems interact [31].

In this study, the number of items within a domain was summed and, if the result was ≥ 2, problems on a domain were considered to be present. Subsequently, we combined the domains and found that the affected number of domains was nonlinearly associated with poor health/wellbeing. Most studies are directed to one of these domains and, if studies are directed to multiple domains, these studies add up to the number of positive items. Here, we have shown that the number of domains with problems is associated with poor health. Therefore, summing the number of positive items without taking into account the additional effect will underestimate the negative consequences for older persons with problems in multiple domains.

### Strengths and limitations

A strength of the present study is the large heterogeneous study population of community- dwelling older people; this adds to the generalizability of the results. Another strength is that health indicators were measured with validated questionnaires by research nurses during home visits, increasing the reliability and the completeness of the measurements. Moreover, participants were followed for one year, which allowed studying the association between the 4 health domains and poor health indicators at 12-months follow-up.

A possible limitation is that poor MMSE-score is associated with death and nursing home admission, leading to selective dropout. This could be an explanation for the lack of association of the number of domains with MMSE-score at 12 months of follow up. Also, since the number of domains with problems at baseline was associated with an increased risk to dropout at 12 months due to death or admission to a nursing home, this group of older people might be the most vulnerable for deterioration in health. Therefore, the associations found in the present study might be an underestimation of the true effect.

Another limitation is that the studied problems on the four domains measure—to greater or lesser extent—the same constructs as some of the negative health indicators (functional domain—GARS, mental domain—MMSE and GDS-15 and social domain—Loneliness Scale of De Jong Gierveld). However, we added also the EQ-5D and GP-contact time as indicators of general health, which also show a strong association with all of the domains.

### Implications

The present study shows that the number of domains with problems at baseline is associated with more disability, impaired cognition, depressive symptoms, feelings of loneliness, poorer HRQoL and more GP contact time, and that having problems on 4 domains is associated with an additional negative impact on these health indicators at baseline, and on GP contact time at follow-up. Since older people with problems on 4 domains have high care needs and poor health/wellbeing, these individuals could be candidates for integrated care. However, the provision of integrated care interventions requires careful identification of older people who will benefit from such an intervention. The method described in this study, i.e. to identify problems on 4 separate health domains and subsequently sum the number of problems per domain, could be a promising instrument to identify older people who are likely to benefit from integrated care interventions; moreover, only a 21-item questionnaire is needed to collect all the required information. Further research is needed to develop integrated primary care interventions for older people with problems on the 4 domains as described here.

### Conclusion

Having problems on four health domains is associated with the poorest scores on health indicators and has an additional negative effect on disability, cognitive function, depressive symptoms, loneliness, HRQoL, and contact time with the GP at baseline. At follow-up, the number of domains with problems is associated with poorer scores; problems on all 4 domains resulted in an additional increase in GP contact time. Problems on all 4 health domains have a nonlinear association with poor health/wellbeing, which may reflect the complexity of older individuals with problems on these domains.

## Supporting Information

S1 File ISCOPE screening questionnaire. (DOC)

S2 File Measuring complex problems in older people: clinimetric properties of a postal screening questionnaire. Unpublished related manuscript.
(DOCX)

S1 Table Prevalence of the 16 possible combinations of the domains with problems at baseline (n = 2681). (DOC)

S2 Table Baseline characteristics and scores on health indicators, for participants with and without problems on each domain (n = 2681). Data are numbers (%) or medians [IQR]. p-trend values were obtained with chi-square tests (categorical data) or linear regression analysis(continuous data). GARS = Groningen Activities Restriction Scale, GDS-15 = Geriatric Depression Scale, MMSE = Mini-Mental State Examination, EQ-5D = Euroqol-5D, GP = General practitioner. Individuals can have problems on more than one domain, therefore the number of participants adds up to a higher number than the study population. ‡ GP contact information was available for 1473 participants
(DOC)

S3 Table Association of the individual domains and the scores on health indicators at 12 months of follow-up (t12). *linear regression analyses modelled on the effect of the individual domains on the score on the health-indicator at after 12 months of follow up (adjusted for age, sex and baseline score of the health indicator). ¥ Loneliness Scale of De Jong Gierveld
(DOC)

## References

[1] Grau L, Kovner C. Comorbidity, age, and hospital use among elderly Medicare patients. J Aging Health 1991; 3: 352–367. 10170759 10.1177/089826439100300303

[2] Marengoni A, von Strauss E, Rizzuto D, Winblad B, Fratiglioni L. The impact of chronic multimorbidity and disability on functional decline and survival in elderly persons. A community-based, longitudinal study. J Intern Med. 2009; 265: 288–295. JIM2017 [pii]; 10.1111/j.1365-2796.2008.02017.x 19192038

[3] Glynn LG, Valderas JM, Healy P, Burke E, Newell J, Gillespie P, et al The prevalence of multimorbidity in primary care and its effect on health care utilization and cost. Fam Pract. 2011; 28: 516–523. cmr013 [pii]; 10.1093/fampra/cmr013 21436204

[4] Fried LP, Bandeen-Roche K, Kasper JD, Guralnik JM. Association of comorbidity with disability in older women: the Women's Health and Aging Study. J Clin Epidemiol. 1999; 52: 27–37. S0895435698001243 [pii]. 9973071 10.1016/s0895-4356(98)00124-3

[5] Penninx BW, Geerlings SW, Deeg DJ, van Eijk JT, van TW, Beekman AT. Minor and major depression and the risk of death in older persons. Arch Gen Psychiatry. 1999; 56: 889–895. 10530630 10.1001/archpsyc.56.10.889

[6] Beekman AT, Penninx BW, Deeg DJ, de Beurs E, Geerlings SW, van Tilburg W. The impact of depression on the well-being, disability and use of services in older adults: a longitudinal perspective. Acta Psychiatr Scand. 2002; 105: 20–27. 12086221 10.1034/j.1600-0447.2002.10078.x

[7] Lyness JM, Kim J, Tang W, Tu X, Conwell Y, King DA et al The clinical significance of subsyndromal depression in older primary care patients. Am J Geriatr Psychiatry. 2007; 15: 214–223. 01.JGP.0000235763.50230.83 [pii]; 10.1097/01.JGP.0000235763.50230.83 17213374

[8] Unutzer J, Patrick DL, Diehr P, Simon G, Grembowski D, Katon W. Quality adjusted life years in older adults with depressive symptoms and chronic medical disorders. 2002; Int Psychogeriatr 12: 15–33. 10.1017/s1041610200006177 10798451

[9] Van der Weele GM, Gussekloo J, De Waal MW, De Craen AJ, Van der Mast RC. Co-occurrence of depression and anxiety in elderly subjects aged 90 years and its relationship with functional status, quality of life and mortality. Int J Geriatr Psychiatry. 2009; 24: 595–601. 10.1002/gps.2162 19031476

[10] Bootsma-van der Wiel A, Gussekloo J, De Craen AJ, Van Exel E, Bloem BR, Westendorp RG. Common chronic diseases and general impairments as determinants of walking disability in the oldest-old population. J Am Geriatr Soc. 2002; 50: 1405–1410. jgs50363 [pii]. 12164998 10.1046/j.1532-5415.2002.50363.x

[11] Perissinotto CM, Stijacic C, I, Covinsky KE. Loneliness in older persons: a predictor of functional decline and death. Arch Intern Med. 2012; 172: 1078–1083. 1188033 [pii]; 10.1001/archinternmed.2012.1993 22710744 PMC4383762

[12] Bekhet AK, Zauszniewski JA. Mental health of elders in retirement communities: is loneliness a key factor? Arch Psychiatr Nurs. 2012; 26: 214–224. S0883-9417(11)00129-4 [pii]; 10.1016/j.apnu.2011.09.007 22633583 PMC3361680

[13] Drageset J, Espehaug B, Kirkevold M. The impact of depression and sense of coherence on emotional and social loneliness among nursing home residents without cognitive impairment—a questionnaire survey. J Clin Nurs. 2012; 21: 965–974. 10.1111/j.1365-2702.2011.03932.x 22250600

[14] Vinkers DJ, Stek ML, Gussekloo J, Van der Mast RC, Westendorp RG. Does depression in old age increase only cardiovascular mortality? The Leiden 85-plus Study. Int J Geriatr Psychiatry. 2004; 19: 852–857. 10.1002/gps.1169 15352142

[15] Barry LC, Allore HG, Bruce ML, Gill TM (2009) Longitudinal association between depressive symptoms and disability burden among older persons. J Gerontol A Biol Sci Med Sci 64: 1325–1332. glp135 [pii]; 10.1093/gerona/glp135 19776217 PMC2773818

[16] Hardy SE, Allore HG, Guo Z, Dubin JA, Gill TM. The effect of prior disability history on subsequent functional transitions. J Gerontol A Biol Sci Med Sci. 2006; 61: 272–277. 61/3/272 [pii]. 16567377 10.1093/gerona/61.3.272

[17] Poot AJ, den Elzen WP, Blom JW, Gussekloo J. Level of satisfaction of older persons with their general practitioner and practice: role of complexity of health problems. PLoS One. 2014; 9: e94326 10.1371/journal.pone.0094326;PONE-D-13-49650 [pii]. 24710557 PMC3978057

[18] Kempen GI, Miedema I, Ormel J, Molenaar W. The assessment of disability with the Groningen Activity Restriction Scale. Conceptual framework and psychometric properties. Soc Sci Med. 1996; 43: 1601–1610. S0277953696000573 [pii]. 8961404 10.1016/s0277-9536(96)00057-3

[19] Bootsma-van der Wiel A, Gussekloo J, De Craen AJ, Van Exel E, Knook DL, Lagaay AM, et al Disability in the oldest old: "can do" or "do do"? J Am Geriatr Soc. 2001; 49: 909–914. jgs49181 [pii]. 11527482 10.1046/j.1532-5415.2001.49181.x

[20] Folstein MF, Folstein SE, McHugh PR. "Mini-mental state". A practical method for grading the cognitive state of patients for the clinician. J Psychiatr Res. 1975; 12: 189–198. 0022-3956(75)90026-6 [pii]. 10.1016/0022-3956(75)90026-6 1202204

[21] Weeks SK, McGann PE, Michaels TK, Penninx BW. Comparing various short-form Geriatric Depression Scales leads to the GDS-5/15. J Nurs Scholarsh. 2003; 35: 133–137. 12854293 10.1111/j.1547-5069.2003.00133.x

[22] Gussekloo J, Westendorp RG, Remarque EJ, Lagaay AM, Heeren TJ, Knook DL. Impact of mild cognitive impairment on survival in very elderly people: cohort study. BMJ. 1997; 315: 1053–1054. 9366730 10.1136/bmj.315.7115.1053 PMC2127688

[23] de Jong-Gierveld J, Kamphuls F. The Development of a Rasch-Type Loneliness Scale. Applied Psychological Measurement. 1985; 9: 289–299.

[24] The EuroQol Group. EuroQol—a new facility for the measurement of health-related quality of life. Health Policy. 1990 16: 199–208. 10109801 10.1016/0168-8510(90)90421-9

[25] Fried LP, Ferrucci L, Darer J, Williamson JD, Anderson G. Untangling the concepts of disability, frailty, and comorbidity: implications for improved targeting and care. J Gerontol A Biol Sci Med Sci. 2004; 59: 255–263. 15031310 10.1093/gerona/59.3.m255

[26] Karlamangla AS, Singer BH, McEwen BS, Rowe JW, Seeman TE. Allostatic load as a predictor of functional decline. MacArthur studies of successful aging. J Clin Epidemiol. 2002; 55: 696–710. S0895435602003992 [pii]. 12160918 10.1016/s0895-4356(02)00399-2

[27] Seeman TE, McEwen BS, Rowe JW, Singer BH. Allostatic load as a marker of cumulative biological risk: MacArthur studies of successful aging. Proc Natl Acad Sci U S A. 2001; 98: 4770–4775. 10.1073/pnas.081072698;081072698 [pii]. 11287659 PMC31909

[28] Tinetti ME, McAvay GJ, Chang SS, Newman AB, Fitzpatrick AL, Fried TR, et al Contribution of multiple chronic conditions to universal health outcomes. J Am Geriatr Soc. 2011; 59: 1686–1691. 10.1111/j.1532-5415.2011.03573.x 21883118 PMC3622699

[29] Aarts S. Co-presence of disability and multimorbidity in health related functioning: temporary or peristent? results from a longitudinal cohort study. In: Multimorbidity in general practice: adverse health effects and innovative research strategies; 2012 pp. 69–81. Available: http://pub.maastrichtuniversity.nl/f25da4bb-f28b-4af3-8012-666fd004ad4f

[30] Lutomski JE, Baars MA, Boter H, Buurman BM, den Elzen WP, Jansen AP, et al [Frailty, disability and multi-morbidity: the relationship with quality of life and healthcare costs in elderly people]. Ned Tijdschr Geneeskd.2014; 158: A7297 25204442

[31] Huyse FJ, Stiefel FC, de Jonge P. Identifiers, or "red flags," of complexity and need for integrated care. Med Clin North Am. 2006; 90: 703–712. 16843770 10.1016/j.mcna.2006.05.003

