# Supplementary material for: Consequences of Interaction of Functional, Somatic, Mental and Social Problems in Community-Dwelling Older People
Source: PLoS One. 2015 Apr 21;10(4):e0121013. doi: 10.1371/journal.pone.0121013 (PMC4405543; doi:10.1371/journal.pone.0121013)
Supplement: S1 File — (DOC) [file pone.0121013.s001.doc]

**S1 File**

**ISCOPE screening questionnaire**

**Daily life abilities**

These first questions relate to how you function/manage day to day life

You may be helped in these activities by aids such as a stick, walking frame, or wheelchair.

1. Can you, without help from anyone else, do the shopping?

*Yes (0 pts.)/ No (1 pts.)*

2. Can you, without help from anyone else, walk outdoors?

*Yes (0 pts.)/ No (1 pts.)*

3. Can you, without help from anyone else, dress and undress yourself?

*Yes (0 pts.)/ No (1 pts.)*

4. Can you, without help from anyone else, go to the toilet?

*Yes (0 pts.)/ No (1 pts.)*

5. Can you manage your finances yourself (collect your money, pay your bills)?

*Yes (0 pts.)/ No (1 pts.)*

6. How well would you say you cope with your general day to day life?

*Well (0 pts.)/So-so (1 pts.)/Not at all well (1 pts.)*

**Health and illness**

7. Which mark would you give your physical fitness?

*1-6 (1 pts.)/ 7-10 (0 pts.)*

*Not at all fit Very fit*

8. Do you experience day-to-day problems due to poor eyesight (even if you wear glasses or contact lenses)?

*Yes (1 pts.) / No (0 pts.)*

9. Do you experience day-to-day problems due to poor hearing (even if you wear a hearing aid)?

*Yes (1 pts.) / No (0 pts.)*

10. Have you lost weight (more than 6 kg) in the last 6 months unintentionally?

*Yes (1 pts.) / No (0 pts.)*

11. Are you using more than 4 different kinds of medicine at the moment?

*Yes (1 pts.) / No (0 pts.)*

12. Have you had a fall in the last month?

*Yes (1 pts.) / No (0 pts.)*

13. Have you been admitted to the hospital in the last 6 months?

*Yes (1 pts.) / No (0 pts.)*

**Psychological functioning**

14. Do you feel you have memory complaints?

*Yes (1 pts.)/Sometimes (1 pts.)/ No (0 pts.)*

15. Have you recently felt sad or depressed?

*Yes (1 pts.)/Sometimes (1 pts.)/ No (0 pts.)*

16. Have you recently felt nervous or anxious?

*Yes (1 pts.)/Sometimes (1 pts.)/ No (0 pts.)*

17. Do you feel pretty worthless at the moment?

*Yes (1 pts.)/Sometimes (1 pts.)/ No (0 pts.)*

**Social functioning**

18. Do you feel that your life is empty?

*Yes (1 pts.)/Sometimes (1 pts.)/ No (0 pts.)*

19. Do you feel the lack of a close friend?

*Yes (1 pts.)/Sometimes (1 pts.)/ No (0 pts.)*

20. Do you feel left alone sometimes?

*Yes (1 pts.)/Sometimes (1 pts.)/ No (0 pts.)*

21. Do you feel there are enough people with whom you feel a close connection?

*Yes (1 pts.)/Sometimes (1 pts.)/ No (0 pts.)*
