# Supplementary material for: Consequences of Interaction of Functional, Somatic, Mental and Social Problems in Community-Dwelling Older People
Source: PLoS One. 2015 Apr 21;10(4):e0121013. doi: 10.1371/journal.pone.0121013 (PMC4405543; doi:10.1371/journal.pone.0121013)
Supplement: S2 File — Unpublished related manuscript. (DOCX) [file pone.0121013.s002.docx]

**Measuring complex problems in older people: clinimetric properties of a postal screening questionnaire**

Anne H. van Houwelingen MD, [a.h.van_houwelingen@lumc.nl](mailto:a.h.van_houwelingen@lumc.nl)

Wendy P.J. den Elzen PhD, [w.p.j.den_elzen@lumc.nl](mailto:w.p.j.den_elzen@lumc.nl)

Margot Heijmans MD, [mheijmans@shg.nl](mailto:mheijmans@shg.nl)

Jacobijn Gussekloo MD PhD, [jgussekloo@lumc.nl](mailto:jgussekloo@lumc.nl)

Jeanet W. Blom MD PhD, [j.w.blom@lumc.nl](mailto:j.w.blom@lumc.nl)

Department of Public Health and Primary Care, Leiden University Medical Center, Leiden, the Netherlands

Number of text words: 3733 Number of tables: 5

Number of figures: 2 Number of appendices: 2

Number of references: 31

**Corresponding author:**

Dr. J.W. Blom

Dept. of Public health & Primary care
Leiden University Medical Center

Post zone V-0-P

P.O. Box 9600
2300 RC Leiden

The Netherlands

Telephone: 31-71-5268444

Fax: 31-71-5268259

E-mail: j.w.blom@lumc.nl

Keywords: Elderly/aged, measurement, complex problems, clinimetric evaluation, questionnaire

**Abstract**

**Background**

The identification of older persons with complex problems is receiving increasing attention. This study analyzes the clinimetric properties of a new self-administered postal questionnaire to identify complex problems in older persons in primary care.

**Methods**

This cross-sectional study was embedded in the Integrated Systematic Care for Older People (ISCOPE) study. Participants filled out the ISCOPE screening questionnaire, a 21-item questionnaire measuring 4 health domains (functional, somatic, mental, social). To investigate the feasibility of the ISCOPE screening questionnaire, a non-response analysis was carried out and the proportion of patients that completed all items was calculated. Cronbach’s alpha was used to assess internal consistency. Spearman’s rank correlations between the questionnaire and health indicators were used to test the construct validity . Test-retest reliability was examined in a sample of 257 participants who filled out the questionnaire twice within 6 weeks. To investigate its content validity, 16 experts were invited to comment on the questionnaire.

**Results**

Of the 11,479 eligible older people, 7285 (63%) participated. Of these, 7178 (98.6%) completed all 21 items of the ISCOPE screening questionnaire. Internal consistency of the 4 domains and the ISCOPE questionnaire was good for the functional (Cronbach’s alpha 0.81) and social domain (0.70); the other 2 domains had poorer internal consistency (somatic domain 0.52, mental domain 0.63). All 10 hypotheses tested to assess construct validity were confirmed. The test-retest reliability of the ISCOPE questionnaire was 65-94% (i.e. substantial to good). The content validity was good: most experts considered all items to be relevant, but proposed 11 items to be added to the questionnaire.

**Conclusion**

The ISCOPE screening questionnaire seems an appropriate instrument to identify older people with complex problems in general practice. Generally, the feasibility, internal consistency, construct validity and test-retest reliability were reasonable. Further research should investigate the responsiveness, generalizability, and predictive validity of this questionnaire.

**Trial registration**

Netherlands Trial Register: NTR1946.

**Background**

The rapidly aging population in the Western world{FormattingCitation}[1,2] has important consequences for primary care, because it faces an increase in home-dwelling older persons with complex problems. The identification of older persons with complex problems, followed by geriatric assessment and long-term management, can improve the daily function of these individuals and may help prevent hospital admission and mortality[3-7]. However, the success of such programs largely depends on the identification of older persons with complex problems. Such identification may distinguish between older persons who will benefit from a pro-active approach[5,8] and vital older persons for whom usual care is generally sufficient. Therefore, this study aimed to develop a simple measure to identify older people with complex problems who will benefit from a pro-active approach in general practice[9,10].

The present study was embedded in the Integrated Systematic Care for Older PEople (ISCOPE) study, which investigates the (cost)-effectiveness of identification of older persons with complex problems followed by a proactive integrated care plan for these persons in general practice.

For this study we developed the ISCOPE screening questionnaire that: 1) identifies complex problems in older people in line with the definition of the Dutch College of General Practitioners, which defined complex problems as having one or more (health) problems that often interact [9,10], 2) is suitable for older people in general practice, and 3) is discriminative: i.e. that it can be used to distinguish older persons with complex problems from vital older persons. The ISCOPE screening questionnaire consisted of 21 questions (Appendix 1) divided into 4 domains of health (i.e. functional, somatic, mental and social). Since the Dutch College of General Practitioners defines complex problems as multiple problems in multiple health domains, we used a stepped scoring system; we first summed the number of problems within a domain and subsequently added up the number of domains.

This paper reports on the development, feasibility, internal consistency, construct validity, test-retest reliability and content validity of the ISCOPE screening questionnaire.

**Methods**

***Development of the ISCOPE screening questionnaire***

Of all items, 15 were derived from the Groningen Frailty Indicator (GFI) (items 1-4, 7-11, 14-16 and 18-20). The GFI is a short 15-item questionnaire to identify frail older people that is often used in a research or clinical setting in the Netherlands [11]. One GFI item was slightly revised: instead of ‘*Do you miss people around you’* we included the question ‘*Do you miss a good friend’*. Item 13 was derived from the Identification of Seniors at Risk (ISAR) tool[12], item 17 from the Geriatric Depression Scale-15 (GDS-15)[13], and item 21 was derived from the Loneliness scale of De Jong-Gierveld et al.[14].

In the ISCOPE questionnaire, the questions refer to what the participants did or how they felt in the week immediately prior to filling in the questionnaire.

*Scoring*

Dichotomous response options were chosen for the functional and somatic domains, and a 3-point Likert scale was chosen for the mental and social domains. A visual analog scale (VAS) score was used for two questions in the functional and somatic domains.

Problems on a domain were considered present when ≥ 2 questions on a domain were responded to positively. Complex problems were considered present when a participant had problems on ≥ 3 domains.

The score per domain was calculated when all items of the domains were completed. The number of domains was summed only when at least 3 domains were completed. The ISCOPE sum score was calculated when all items of the questionnaire were completed.

*Pilot*

The ISCOPE screening questionnaire was first discussed with a sample of older representatives (n=20). The questionnaire was then piloted in 3 general practices (n=556). All older people in these practices received the postal questionnaire. Of these, 369 older people gave informed consent to participate (62%); their general practitioners (GPs) were then asked to rate the complex problems of these individuals. After this pilot, the GPs received feedback on the questionnaire and were interviewed about the content of the questionnaire, its results, and any differences that emerged compared with their own impression.

***Study population***

The present study was embedded in the ISCOPE study which, in people aged > 75 years with complex problems, compared a proactive approach by their GP with usual care (Blom et al. Effectiveness and cost-effectiveness of a proactive, goal-oriented, integrated care model in general practice for older people. A cluster randomized trial: Integrated Systematic Care for Older People: the ISCOPE study; Submitted).

For the present study 7285 participants who filled out the ISCOPE screening questionnaire were selected; all participants provided written informed consent.

The study was approved by the Medical Ethics Committee of the Leiden University Medical Center.

***Measurements***

For logistical reasons, only a random sample of the study population (n=2713; 43%) was visited at home by a research nurse to obtain data on sociodemographic characteristics and to administer additional questionnaires. All participants with complex problems were visited. In addition, a random sample of 60% of the participants with problems on 2 domains of health, and a random sample of 15% of the participants with problems on 1 or 0 domains of health, were visited.

During home visits, data on sociodemographic characteristics, multimorbidity, functional status and life satisfaction were obtained; these data were self-reported. Chronic diseases included self-reported diabetes, heart failure, malignancy, chronic obstructive pulmonary disease (COPD), incontinence, arthritis, osteoporosis, dizziness, lower urinary tract symptoms (LUTS), depression, anxiety, dementia, vision, deafness, fracture, stroke/transient ischemic attack, and myocardial infarction. A sum score of these listed diseases was calculated.

Competence in basic (BADL) and instrumental activities of daily living (IADL) was measured with the Groningen Activities Restriction Scale (GARS) [15]. The GARS is a questionnaire that assesses disabilities in competence in BADL and IADL [15,16]. Questions were phrased: ‘*Can you fully independently*,…?’. The items of the GARS were classified into BADL or IADL items in accordance with Bootsma-van der Wiel et al. [16]. A sum score was calculated and ranged from 18 (competent in all activities) to 72 (unable to perform any activity without help).

Global cognitive function was assessed with the Mini-Mental State Examination (MMSE) with scores ranging from 0-30 (=optimal)[17]. The Geriatric Depression Scale-15 (GDS-15) was used to assess depressive symptoms; higher scores on the GDS-15 indicate more depressive symptoms[13]. The Loneliness scale of De Jong Gierveld et al. was used to assess feelings of loneliness, with a higher score indicating more severe loneliness (range 0-11)[14]. The GDS-15 and the Loneliness scale were restricted to those individuals with an MMSE score of ≥ 19.

Life satisfaction was assessed with the Cantril ladder[18] (a VAS on perceived quality of life ranging from 1-10)

Prior to randomization the GPs received a list of all patients aged ≥ 75 years in their practices and were asked to indicate which patients were, in their opinion, ‘vulnerable’ individuals.

The number of GP contacts in the year before the start of the study was derived from the electronic patient records of the participants, including the number of consultations, telephone consultations and home visits.

***Analyses***

*Feasibility*

We compared responders and non-responders using patient data from one rural and one city GP practice (total of 629 patients), both of which had participated in the ISCOPE study. Anonymous data from the electronic patient records were available for participants and non-participants. A comparison was made of sociodemographic data, diseases (ICPC codes), medication, use of care, and the GP’s appraisal of vulnerability.

In addition, the proportion of participants of the total study population who completed all items of the questionnaire and completed at least 3 domains of the questionnaire, and the proportion of participants who received help with filling out the questionnaire, were calculated.

*Internal consistency*

The internal consistency for each domain and for the complete ISCOPE screening questionnaire was investigated. First, the internal consistency of the domains and the ISCOPE screening questionnaire was examined using Cronbach’s alpha coefficient. A Cronbach’s alpha coefficient > 0.7 was considered acceptable [19]. Subsequently, the average inter-item correlations were calculated, as these are independent from the scale length. We considered an average inter-item correlation of 0.20-0.70 to be satisfactory. Finally, item-rest correlations between the individual items and the sum of the remaining items of a domain were calculated.

To explore whether there was a mutual correlation between the various items of the four domains, or with items within other domains, we constructed a correlation matrix of all items and a correlation matrix of the items with problems on the individual domains.

*Construct validity*

Construct validity was evaluated by defining hypotheses based on previous literature [20-24] or on clinical experience. The following hypotheses were tested:

1. There is a positive correlation with age.
2. There is a positive correlation with score on the GARS.
3. There is a positive correlation with the number of contacts with the GP.
4. There is a negative correlation with the score on Cantril’s ladder.
5. There is a positive correlation with the number of chronic diseases.
6. There is a positive correlation with the GP’s perspective on vulnerability.
7. The correlation between the functional domain and the GARS is higher than that between the other three domains and the GARS.
8. The correlation between the somatic domain and the number of chronic diseases is higher than that between the other three domains and the number of chronic diseases.
9. The correlation between the mental domain and the GDS-15 is higher than that between the other three domains and the GDS-15.
10. The correlation between the social domain and the Loneliness scale of De Jong Gierveld et al. is higher than that between the other three domains and the Loneliness scale of De Jong Gierveld et al.

These hypotheses were tested with Spearman’s correlation coefficient since the data were not normally distributed. The construct validity was considered satisfactory when ≥ 75% of our hypotheses were correct.

*Test-retest reliability*

Test-retest reliability refers to the reproducibility of measurements using the same instruments over time. A subgroup of the participants (n=494) from 5 general practices received the ISCOPE screening questionnaire twice (12 months after study inclusion) within a period of 2-4 weeks; this period was considered long enough to ensure that participants would not remember their first responses and short enough to ensure that the situation of the participant had not changed substantially. An item was added to the second questionnaire to assess whether participants considered themselves ‘stable’ during the period between the two questionnaires. Only data of participants who considered themselves to be stable were used.

Participants who received help with filling out the first **or** second questionnaire were also excluded; however, participants who received help with **both** questionnaires were included. For dichotomous response options, we used the percentage agreement and kappa statistic. For categorical response options, the intraclass correlation coefficient (ICC) and Bland and Altman plots were used. A kappa of 0.61 and an ICC of 0.70 were considered to be satisfactory[25,26]. Mean differences between the two measurements were calculated with limits of agreement according to Bland and Altman[27].

A p-value of <0.05 was considered statistically significant. Data were analyzed with SPSS 20.0 for Windows and VassarStats website for statistical computation.

*Content validity*

Content validity is defined as the degree to which the content of a measurement instrument is an adequate reflection of the construct to be measured [28]. Therefore, a panel of 12 trained professionals in the geriatric field (5 GPs with special interest in elderly care, 3 elderly care physicians and 4 geriatricians) as well as 3 male and 1 female older volunteers, were asked to assess if all items were relevant and whether the questionnaire was sufficiently comprehensive. The professionals in the geriatric field were selected based on their experience in this area and familiarity with identification of complex problems in older persons. All received an email with a web link to a website, which provided them with the definition of complex problems according to the statement of the Dutch College of General Practitioners[10] and its 4 dimensions (Panel 1)., and the various questions of the ISCOPE questionnaire per domain They were asked to evaluate the relevance of each of the items per domain, to suggest items to add to a domain (only if they considered one of the domains incomplete), and to indicate if one of the original items should be replaced by one of their suggested items per domain.

**Results**

*Adjustments to the ISCOPE screening questionnaire before conducting the study*

The 20 older representatives that commented on the ISCOPE screening questionnaire before the study largely agreed with its contents. All of them filled out the questionnaire as though they were a participant in the study; their main comment concerned the spelling and/or formulation of some of the items. Based on these comments, the questionnaire was slightly adjusted.

This revised version of the questionnaire was then piloted in three general practices. The three GPs were asked to comment on the questionnaire. In general, they agreed that the questionnaire yielded results that were applicable in their practice, and that the older persons with complex problems as identified with the questionnaire were eligible for integrated care.

*Study population*

Of the 12,066 registered persons in the 59 practices, 11,479 were eligible. The overall response rate was 63%; 7285 older persons participated in the present study. The screening questionnaire was missing for 7 persons, resulting in a study population of 7278 older persons. One third of the study population (n=2713) was interviewed at home (Figure 1).

Table 1 presents the characteristics of the study population: median age was 81 (IQR 77-85) years and there were more females (61.4%) than males (38.6%). Of all participants, 26.4% had complex problems according to the ISCOPE screening questionnaire.

*Feasibility*

The response in the two practices for non-response analysis was 57%. Non-responders were more often women (41.9% vs 33.9%, p=0.043), were older [median 81 (IQR 78-85) years vs. 80 (77-83) years; p=0.01], were appraised more often as ‘vulnerable’ by the GP (25.1% vs. 15.6%; p=0.025) and received more home visits from their GP (44.6% vs 36.9%; p=0.049). In addition, cognitive decline or problems with vision were more often registered (Blom et al. Effectiveness and cost-effectiveness of a proactive, goal-oriented, integrated care model in general practice for older people. A cluster randomized trial: Integrated Systematic Care for Older People – the ISCOPE study; Submitted).

Of the 7285 participants included in this study, 7178 (98.6%) completed all items of the ISCOPE screening questionnaire. One third of the population (2237, 31%) was assisted by a relative (n=1395, 19%) or a research nurse (n=842, 12%). Compared with participants who did not need assistance, those with assistance were generally older, more often female, and more often had complex problems (all p <0.001). Based on the experience of the individual research nurses, it took 7-8 min to administer the questionnaire verbally. When the research nurses attended the completion of the questionnaire by the participant, the completion also took 7-8 min.

*Internal consistency*

The ISCOPE screening questionnaire had an internal consistency of 0.82. The internal consistency was 0.81 for the functional domain, 0.51 for the somatic domain, 0.63 for the mental domain and 0.70 for the social domain. Because the internal consistency of the somatic domain was low, we explored whether items of the somatic domain were related to other domains. The items of the somatic domain had a low correlation with the other domains (range 0.045-0.176); however, the *physical fitness* item correlated (range 0.40-0.47) with 3 items of the functional domain (*shopping, walk outdoors and cope with general day-to-day life)*.

The mental domain had an internal consistency of 0.63. When we omitted the *memory complaints* item, the internal consistency increased to 0.69.

The average inter-item correlation was 0.372 and item-rest correlations ranged from 0.413-0.567. All domains had moderate mutual correlations: these ranged from 0.21 between the functional and social domain to 0.52 between the mental and social domain (Table 2).

*Construct validity*

Table 3 lists all hypotheses and the corresponding correlations. All 10 hypotheses were confirmed (100%).

*Test-retest reliability*

Participants from 5 general practices (n=494) were invited for a test-retest analysis. We excluded participants who refused to fill out the second questionnaire (n=130), participants for whom the second questionnaire was missing (n=5), participants who reported a change (n=66), and participants who filled out the two questionnaires in two different ways (i.e. with and without assistance) (n=36). This resulted in a study population of 257 participants (52%) for the test-retest analysis. Table 4 presents the results of the first and second ISCOPE questionnaire with a median time interval between the two tests of 6 (IQR 5-7) weeks. The unadjusted agreement percentage of complex problems was 91% with a kappa of 0.71. The median number of domains with problems was the same during the first and second measurement (median 1 domain, interquartile range (IQR) 0-2 for both measurements). The agreement for the number of domains with problems was 0.76 (linear weighted kappa).

The functional domain had the highest test-retest reliability (unadjusted agreement 94%, kappa 0.81). Agreement and kappa for the other domains was 84% and 0.68 for the mental domain, 86% and 0.71 for the somatic domain, and 88% and 0.69 for the social domain, respectively. For the individual items, agreement ranged from 84-99%. Kappas ranged from 0.42 to 0.85. The *falls* item and the *close connection* item had moderate kappas, but relatively high percentages of agreement, because of the low prevalence[29].

During both the first and second measurements, the median score for the ISCOPE sum score remained the same and the reliability was high (ICC 0.92 [95% CI 0.90-0.94]). Figure 2 shows the differences between the two measurements of the ISCOPE sum score against their means[27].

*Content validity*

Panel 1 presents the definitions of the construct *complex problems* and the 4 domains which were presented to the experts. Most experts considered the items of the ISCOPE screening questionnaire to be relevant for the domain to be measured (Table 5). However, the experts did not find the questionnaire totally comprehensive and suggested to add 30 items (7 to the functional domain, 13 to the somatic domain, 3 to the mental domain, and 7 to the social domain). The experts indicated that 11 of the original items could be replaced. Appendix 2 shows the items that the experts suggested should be added and those that they recommended to be replaced.

**Discussion**

The aim of this study was to describe the clinimetric properties of the ISCOPE screening questionnaire, which aims to identify community-dwelling older people with complex problems by postal screening. Complex problems were defined as a combination of problems on 3 or 4 health domains (functional, somatic, mental and social) of the ISCOPE screening questionnaire. Over 25% of our population was classified as having complex problems. After investigating the feasibility, internal consistency, construct validity test-retest reliability and content validity of the questionnaire, all these clinimetric properties proved to be reasonable.

The items within the health domains were based on a clinical understanding of the problems belonging to each domain*.* Therefore, the domains are not totally comprehensive and this explains the slightly low internal consistencies. This was confirmed by the comments of the experts about the content validity of the questionnaire, i.e. they considered most of the items to be relevant, but suggested adding 11 items that they considered to be missing.

With regard to test-retest reliability, the results show strong to complete agreement between the first and second questionnaire.

*Strengths and limitations*

The present study has some major strengths. First, it was embedded in the ISCOPE study which is a pragmatic randomized trial with few exclusion criteria. This enabled us to include a very heterogeneous community-dwelling older population, which supports the generalizability of the results. Another strength is the stepped scoring system (i.e. summing the number of problems within a domain and subsequently adding up the number of domains), instead of summing the number of positive items, which is a common scoring system for other instruments, such as the GFI. Our system better reflects the combination of health problems that determine complex problems in older people and is in line with the statement of the Dutch College of General Practitioners on complex problems in older people[10]. As far as we know, we are the first to use such as scoring system to identify older people with complex problems.

A limitation of this study is that, for logistical reasons, the expert consultations (and their ideas) about the ISCOPE screening questionnaire could not take place before the measurements were made and, therefore, could not be included in this study. However, the experts’ suggestions will be used to revise the ISCOPE screening questionnaire for further research; then, this later revised version will be tested against the unrevised version of the ISCOPE screening questionnaire.

The moderate response rate may limit the feasibility of our questionnaire for general practice. However, this was measured in a research setting with an intervention and home visits following the questionnaire, and could be higher in a ‘real life’ situation. It is reported that older individuals who do not respond tend to have more complex problems than the respondents[30,31]. In our study, we found that non-responders and responders were largely similar, but non-responders more often had problems with vision and cognitive impairment, were more often appraised as ‘vulnerable’ by their GP and received more home visits. This indicates that these older people may indeed have more complex problems than responders.

Of the responders, about a third received help with completing the questionnaire. Most of these older persons were assisted by a relative, which will probably be the case when the questionnaire is implemented in general practice; the remainder was assisted by a research nurse. Although this resulted in very few missing values, the question arises whether a GP (or practice staff) will have sufficient time to perform such a follow-up in general practice.

**Conclusion**

The ISCOPE screening questionnaire is a promising self-report instrument to identify older individuals with complex problems in the general population. However, additional studies on the responsiveness, generalizability, construct and predictive validity of the ISCOPE screening questionnaire are needed. Moreover, the ISCOPE screening questionnaire may be further improved based on the suggestions of the experts as described in this study. In addition, since this questionnaire was developed to identify older persons with complex problems who were likely to benefit most from integrated geriatric care, we need to investigate whether older persons with complex problems (as identified with this questionnaire) indeed show poor scores on health outcomes and whether they actually benefit most from integrated geriatric care.

**Acknowledgments**

**Authors’ contributions:**

*Guarantor:* JW Blom had full access to all of the data in the study and takes responsibility for the integrity of the data and the accuracy of the data analysis.

*Study concept and design*: JW Blom, WPJ den Elzen, J Gussekloo

*Acquisition of data*: AH van Houwelingen, WPJ den Elzen, M Heijmans, J Gussekloo, JW Blom.

*Analysis and interpretation of data*: AH van Houwelingen, WPJ den Elzen, M Heijmans, J Gussekloo, JW Blom.

*Drafting of the manuscript*: AH van Houwelingen, WPJ den Elzen, J Gussekloo, JW Blom.

*Critical revision of the manuscript for important intellectual content*: AH van Houwelingen, WPJ den Elzen, M. Heijmans, J Gussekloo, JW Blom,

*Obtained funding*: JW Blom and J Gussekloo.

**Copyright**

The Corresponding Author has the right to grant on behalf of all authors and does grant on behalf of all authors, a worldwide licence to the Publishers and its licensees in perpetuity, in all forms, formats and media (whether known now or created in the future), to i) publish, reproduce, distribute, display and store the Contribution, ii) translate the Contribution into other languages, create adaptations, reprints, include within collections and create summaries, extracts and/or, abstracts of the Contribution, iii) create any other derivative work(s) based on the Contribution, iv) to exploit all subsidiary rights in the Contribution, v) the inclusion of electronic links from the contribution to third party material where-ever it may be located; and, vi) licence any

third party to do any or all of the above.

**Competing interests:** None declared.

**Funding/Support:** This study was funded by ZonMw, the Netherlands, Organization for Health Research and Development: Project no. 311060201.

**Role of the Sponsor:** The sponsor had no role in the design and conduct of the study; collection, management, analysis, and interpretation of the data; and preparation, review, or approval of the manuscript.

**Ethical approval**

This study was approved by the Medical Ethical Committee of Leiden University Medical Center in 2009.

**Trial registration**

Netherlands Trial Register: NTR1946.

**References**

1. Christensen K, Doblhammer G, Rau R, Vaupel JW: **Ageing populations: the challenges ahead.** *Lancet* 2009, **374:** 1196-1208.

2. Lutz W, Sanderson W, Scherbov S: **The coming acceleration of global population ageing.** *Nature* 2008, **451:** 716-719.

3. Stuck AE, Siu AL, Wieland GD, Adams J, Rubenstein LZ: **Comprehensive geriatric assessment: a meta-analysis of controlled trials.** *Lancet* 1993, **342:** 1032-1036.

4. Stuck AE, Aronow HU, Steiner A, Alessi CA, Bula CJ, Gold MN *et al*.: **A trial of annual in-home comprehensive geriatric assessments for elderly people living in the community.** *N Engl J Med* 1995, **333:** 1184-1189.

5. Boyd CM, Boult C, Shadmi E, Leff B, Brager R, Dunbar L *et al*.: **Guided care for multimorbid older adults.** *Gerontologist* 2007, **47:** 697-704.

6. Melis RJ, van Eijken MI, Teerenstra S, van Achterberg T, Parker SG, Borm GF *et al*.: **A randomized study of a multidisciplinary program to intervene on geriatric syndromes in vulnerable older people who live at home (Dutch EASYcare Study).** *J Gerontol A Biol Sci Med Sci* 2008, **63:** 283-290.

7. Monteserin R, Brotons C, Moral I, Altimir S, San JA, Santaeugenia S *et al*.: **Effectiveness of a geriatric intervention in primary care: a randomized clinical trial.** *Fam Pract* 2010, **27:** 239-245.

8. Reuben DB: **Medical care for the final years of life: "When you're 83, it's not going to be 20 years".** *JAMA* 2009, **302:** 2686-2694.

9. https://[www.nhg.org/dutch-college-general-practitioners](http://www.nhg.org/dutch-college-general-practitioners) . 4-9-2013.

10. NHG-standpunt Toekomstvisie Huisartsenzorg. Huisartsgeneeskunde voor ouderen. NHG, 2007 . 31-5-2007.

11. Steverink S, Slaets JP, Schuurmans H, Van Lis M: **Measuring frailty: developing and testing the GFI (Groningen frailty Indicator).** *Gerontologist* 2001, **41:** 236-237.

12. McCusker J, Bellavance F, Cardin S, Trepanier S: **Screening for geriatric problems in the emergency department: reliability and validity. Identification of Seniors at Risk (ISAR) Steering Committee.** *Acad Emerg Med* 1998, **5:** 883-893.

13. Weeks SK, McGann PE, Michaels TK, Penninx BW: **Comparing various short-form Geriatric Depression Scales leads to the GDS-5/15.** *J Nurs Scholarsh* 2003, **35:** 133-137.

14. de Jong-Gierveld J, Kamphuls F: **The Development of a Rasch-Type Loneliness Scale.** *Applied Psychological Measurement* 1985, **9:** 289-299.

15. Kempen GI, Miedema I, Ormel J, Molenaar W: **The assessment of disability with the Groningen Activity Restriction Scale. Conceptual framework and psychometric properties.** *Soc Sci Med* 1996, **43:** 1601-1610.

16. Bootsma-van der Wiel A, Gussekloo J, De Craen AJ, Van Exel E, Knook DL, Lagaay AM *et al*.: **Disability in the oldest old: "can do" or "do do"?** *J Am Geriatr Soc* 2001, **49:** 909-914.

17. Folstein MF, Folstein SE, McHugh PR: **"Mini-mental state". A practical method for grading the cognitive state of patients for the clinician.** *J Psychiatr Res* 1975, **12:** 189-198.

18. Cantril H: *The pattern of human concern*. Rutgers University Press; 1965.

19. Bland JM, Altman DG: **Cronbach's alpha.** *BMJ* 1997, **314:** 572.

20. Alessi CA, Josephson KR, Harker JO, Pietruszka FM, Hoyl MT, Rubenstein LZ: **The yield, reliability, and validity of a postal survey for screening community-dwelling older people.** *J Am Geriatr Soc* 2003, **51:** 194-202.

21. De Saint-Hubert M, Schoevaerdts D, Cornette P, D'Hoore W, Boland B, Swine C: **Predicting functional adverse outcomes in hospitalized older patients: a systematic review of screening tools.** *J Nutr Health Aging* 2010, **14:** 394-399.

22. Peters LL, Boter H, Buskens E, Slaets JP: **Measurement Properties of the Groningen Frailty Indicator in Home-Dwelling and Institutionalized Elderly People.** *J Am Med Dir Assoc* 2012, **13:** 546-551.

23. Ravaglia G, Forti P, Lucicesare A, Pisacane N, Rietti E, Patterson C: **Development of an easy prognostic score for frailty outcomes in the aged.** *Age Ageing* 2008, **37:** 161-166.

24. Grant RW, Ashburner JM, Hong CC, Chang Y, Barry MJ, Atlas SJ: **Defining Patient Complexity From the Primary Care Physician's Perspective A Cohort Study.** *Ann of Intern Med* 2011, **155:** 797-804.

25. Streiner DL, Norman G.R.: *Health measurement scales. A practical guide to their development and use. 4th ed.* New York: Oxford University press; 2008.

26. **Assessing health status and quality-of-life instruments: attributes and review criteria.** *Qual Life Res* 2002, **11:** 193-205.

27. Bland JM, Altman DG: **Statistical methods for assessing agreement between two methods of clinical measurement.** *Lancet* 1986, **1:** 307-310.

28. Mokkink LB, Terwee CB, Patrick DL, Alonso J, Stratford PW, Knol DL *et al*.: **The COSMIN checklist for assessing the methodological quality of studies on measurement properties of health status measurement instruments: an international Delphi study.** *Qual Life Res* 2010, **19:** 539-549.

29. Feinstein AR, Cicchetti D: **High agreement but low Kappa: I. the problems of two paradoxes.** *J Clin Epidemiol* 1990, **43:** 543-549.

30. Barreto PS: **Participation bias in postal surveys among older adults: the role played by self-reported health, physical functional decline and frailty.** *Arch Gerontol Geriatr* 2012, **55:** 592-598.

31. Bowns I, Challis D, Tong MS: **Case finding in elderly people: validation of a postal questionnaire.** *Br J Gen Pract* 1991, **41:** 100-104.

| **Table 1.** Baseline characteristics of the study population (n=7278) | |
| --- | --- |
| Age (years) | 81 (77-85) |
| Male sex | 2812 (38.6) |
| Number of domains with problems according to the ISCOPE questionnaire | 1 (0-3) |
| Complex problems according to the ISCOPE questionnaire | 1921 (26.4) |
| Data are presented as numbers (%) or median (IQR) | |

| **Table 2.** Mean scores and intercorrelations between the four domains | | | | | | | |
| --- | --- | --- | --- | --- | --- | --- | --- |
|  | | N | Mean | SD | 1* | 2* | 3* |
| 1 | Functional domain | 6 | 0.97 | 1.52 |  |  |  |
| 2 | Somatic domain | 7 | 1.78 | 1.43 | 0.479 |  |  |
| 3 | Mental domain | 4 | 1.55 | 1.31 | 0.349 | 0.410 |  |
| 4 | Social domain | 4 | 1.13 | 1.28 | 0.210 | 0.264 | 0.517 |
| * Pearson’s correlation coefficient | | | | | | | |

| **Table 3.** Construct validity of the ISCOPE screening questionnaire | | | | |
| --- | --- | --- | --- | --- |
| Hypothesis* | | n | Spearman's correlation coefficient | p-value |
|  | Presence of complex problems |  |  |  |
| 1 | Age | 2713 | 0.172 | <0.001 |
| 2 | Disability | 2713 | 0.422 | <0.001 |
| 3 | Health care use | 2713 | 0.196 | <0.001 |
| 4 | Life satisfaction | 2713 | -0.290 | <0.001 |
| 5 | Diseases | 2713 | 0.324 | <0.001 |
| 6 | GP's perspective | 2534 | 0.212 | <0.001 |
|  | Domains |  |  |  |
| 7 | Functional domain |  |  |  |
|  | Functional domain - GARS | 2683 | 0.668 | <0.001 |
|  | Somatic domain - GARS | 2676 | 0.393 | <0.001 |
|  | Mental domain - GARS | 2679 | 0.160 | <0.001 |
|  | Social domain - GARS | 2683 | 0.046 | 0.023 |
| 8 | Somatic domain |  |  |  |
|  | Somatic domain - number of chronic diseases | 2709 | 0.369 | <0.001 |
|  | Functional domain - number of chronic diseases | 2702 | 0.254 | <0.001 |
|  | Mental domain - number of chronic diseases | 2705 | 0.266 | <0.001 |
|  | Social domain - number of chronic diseases | 2708 | 0.144 | <0.001 |
| 9 | Mental domain |  |  |  |
|  | Psychological domain – GDS-15 | 2548 | 0.368 | <0.001 |
|  | Functional domain – GDS-15 | 2544 | 0.288 | <0.001 |
|  | Somatic domain – GDS-15 | 2544 | 0.254 | <0.001 |
|  | Social domain – GDS-15 | 2544 | 0.277 | <0.001 |
| 10 | Social domain |  |  |  |
|  | Social domain - Loneliness Scale | 2542 | 0.535 | <0.001 |
|  | Functional domain - Loneliness Scale | 2542 | 0.071 | <0.001 |
|  | Somatic domain - Loneliness Scale | 2538 | 0.124 | <0.001 |
|  | Mental domain - Loneliness Scale | 2538 | 0.270 | <0.001 |
| GFI= Groningen Frailty Indicator, GARS= Groningen Activities Restriction scale, GDS-15=Geriatric Depression Scale-15  10 hypotheses were tested  1. There is a positive correlation with age  2. There is a positive correlation with score on the GARS  3. There is a positive correlation with the number of contacts with the GP  4. There is a negative correlation with score on Cantril’s ladder  5. There is a positive correlation with the number of chronic diseases  6. There is a positive correlation with GP's perspective on vulnerability  7. The correlation between the functional domain and the GARS is higher than the correlation between the other three domains and the GARS  8. The correlation between the somatic domain and the number of chronic diseases is higher than the correlation between the other three domains and the number of chronic diseases  9. The correlation between the mental domain and the GDS-15 is higher than the correlation between the other three domains and the GDS-15  10. The correlation between the social domain and the Loneliness scale of De Jong Gierveld et al. is higher than the correlation between the other three domains and the Loneliness scale of De Jong Gierveld et al. | | | | |

| **Table 4.** Test-retest reliability of respondents of the ISCOPE screening questionnaire (n=257) | | | | |
| --- | --- | --- | --- | --- |
|  | Time 1 | Time 2 | Agreement | (weighted) Kappa or ICC |
|  |  |  |  |  |
|  | n (%) | n (%) |  |  |
| Age* | 81 (79-85) |  |  |  |
| Male sex | 114 (44.4) |  |  |  |
|  |  |  |  |  |
| **Functional domain** | 48 (18.7) | 51 (19.6) | 94 | 0.812 |
| shopping | 38 (14.8) | 42 (16.3) | 96 | 0.852 |
| walk outdoors | 27 (10.5) | 33 (12.8) | 95 | 0.736 |
| dress and undress | 9 (3.5) | 14 (5.4) | 98 | 0.773 |
| go to the toilet | 1 (0.4) | 2 (0.8) | 99.6 | 0.665 |
| manage finances | 37 (14.4) | 40 (15.6) | 91 | 0.649 |
| cope with your general day to day life | 67 (26.1) | 61 (23.7) | 87 | 0.647 |
|  |  |  |  |  |
| **Somatic domain** | 112 (43.6) | 112 (43.6) | 86 | 0.714 |
| physical fitness | 79 (30.7) | 84 (32.7) | 89 | 0.740 |
| vision | 40 (15.6) | 39 (15.2) | 94 | 0.776 |
| hearing | 64 (24.9) | 57 (22.2) | 90 | 0.730 |
| unintentional weight loss | 11 (4.3) | 7 (2.7) | 98 | 0.770 |
| > 4 different kinds of medicines | 147 (57.2) | 153 (59.9) | 92 | 0.840 |
| falls | 18 (7.0) | 25 (9.7) | 91 | 0.418 |
| hospital admission | 29 (11.3) | 25 (9.7) | 97 | 0.835 |
|  |  |  |  |  |
| **Mental domain** | 104 (40.5) | 106 (41.2) | 84 | 0.678 |
| memory complaints | 125 (48.6) | 120 (46.7) | 86 | 0.727 |
| sad or depressed | 104 (40.5) | 100 (38.9) | 85 | 0.691 |
| nervous or anxious | 74 (28.8) | 74 (28.9) | 82 | 0.564 |
| worthless | 59 (23.0) | 55 (21.4) | 84 | 0.527 |
|  |  |  |  |  |
| **Social domain** | 74 (28.8) | 70 (27.2) | 88 | 0.691 |
| emptiness | 105 (40.9) | 101 (39.3) | 84 | 0.660 |
| lack of a close friend | 76 (29.6) | 73 (28.4) | 85 | 0.631 |
| left alone | 54 (21.0) | 48 (18.7) | 88 | 0.633 |
| close connection to people | 23 (8.9) | 27 (10.5) | 90 | 0.424 |
|  |  |  |  |  |
| **Number of domains with problems*** | 1 (0-2) | 1 (0-2) | 66 | 0.761 |
| **Total score on ISCOPE questionnaire*** | 4 (2-7) | 4 (2-7) | 36 | 0.918 |
| **Complex problems** | 46 (17.9) | 53 (20.6) | 91 | 0.713 |
| * data are presented as median (IQR) |  |  |  |  |

| **Table 5.** Content validity of the ISCOPE screening questionnaire according to the 16 experts. | | | |
| --- | --- | --- | --- |
|  | All questions relevant | Domain complete | Replace items |
|  | n/16 | n/16 | n/16 |
| Functional domain | 13 | 2 | 6 |
| Somatic domain | 14 | 4 | 4 |
| Mental domain | 14 | 8 | 3 |
| Social domain | 14 | 7 | 7 |
|  |  |  |  |

***Panel 1***

***Definitions of the construct ‘Complex problems’ and the four domains of the ISCOPE screening questionnaire which were presented to the expert panel***

**Complex problems**

An older person with complex problems is someone with multiple health problems which interact. Complex problems are considered present when problems exist on three or more out of four domains; functional, somatic, mental and social. Complex problems result in deterioration in quality of life and functional decline.

**Functional domain**

The functional domain contains ADLs (Activities of Daily Living, i.e. basic activities including washing, dressing and undressing) and IADLs (Instrumental activities of Daily Living, i.e. household tasks including cooking, do the shopping, manage finances).

**Somatic domain**

The somatic domain aims to identify somatic problems including co-morbidity, poly-pharmacy and specific geriatric physical problems.

**Mental domain**The mental domain identifies depressive symptoms and cognition problems

**Social domain**

The social domain contains loneliness and reduced social life
