# Supplementary material for: Consequences of Interaction of Functional, Somatic, Mental and Social Problems in Community-Dwelling Older People
Source: PLoS One. 2015 Apr 21;10(4):e0121013. doi: 10.1371/journal.pone.0121013 (PMC4405543; doi:10.1371/journal.pone.0121013)
Supplement: S1 Table — (DOC) [file pone.0121013.s003.doc]

| **S1 Table** Prevalence of the 16 possible combinations of the domains with problems at baseline (n=2681) | | | | |
| --- | --- | --- | --- | --- |
| Problems | | | |  |
| Functional | Somatic | Mental | Social | **n(%)** |
| - | - | - | - | 243 (9) |
| + | - | - | - | 15 (0.6) |
| - | + | - | - | 99 (4) |
| - | - | + | - | 63 (2) |
| - | - | - | + | 36 (1) |
| + | + | - | - | 139 (5) |
| + | - | + | - | 29 (1) |
| + | - | - | + | 14 (1) |
| - | + | + | - | 267 (10) |
| - | + | - | + | 86 (3) |
| - | - | + | + | 185 (7) |
| + | + | + | - | 341 (13) |
| + | - | + | + | 55 (2) |
| + | + | - | + | 69 (3) |
| - | + | + | + | 562 (21) |
| + | + | + | + | 478 (18) |
