# Supplementary material for: Consequences of Interaction of Functional, Somatic, Mental and Social Problems in Community-Dwelling Older People
Source: PLoS One. 2015 Apr 21;10(4):e0121013. doi: 10.1371/journal.pone.0121013 (PMC4405543; doi:10.1371/journal.pone.0121013)
Supplement: S2 Table — Data are numbers (%) or medians [IQR]. p-trend values were obtained with chi-square tests (categorical data) or linear regression analysis(continuous data). GARS = Groningen Activities Restriction Scale, GDS-15 = Geriatric Depression Scale, MMSE = Mini-Mental State Examination, EQ-5D = Euroqol-5D, GP = General practitioner. Individuals can have problems on more than one domain, therefore the number of participants adds up to a higher number than the study population. ‡ GP contact information was available for 1473 participants (DOC) [file pone.0121013.s004.doc]

| **S2 Table** Baseline characteristics and scores on health indicators, for participants with and without problems on each domain (n=2681) | | | | | | | | | | | | | | | |
| --- | --- | --- | --- | --- | --- | --- | --- | --- | --- | --- | --- | --- | --- | --- | --- |
|  | Domain | | | | | | | | | | | | | | |
|  | **Functional** | |  |  | **Somatic** | |  |  | **Mental** | |  |  | **Social** | |  |
|  | Problems | No problems | p |  | Problems | No problems | p |  | Problems | No problems | p |  | Problems | No problems | p |
|  | n=1140 | n=1541 |  |  | n=2041 | n=640 |  |  | n=1980 | n=701 |  |  | n=1485 | n=1196 |  |
| Age | 84 (80;89) | 81 (79;85) | <0.001 |  | 83 (79;87) | 81 (78;85) | <0.001 |  | 82 (79;87) | 82 (78;86) | 0.020 |  | 82 (79;87) | 82 (78;86) | 0.054 |
| Male sex | 281 (24.5) | 569 (36.9) | <0.001 |  | 634 (31.1) | 216 (33.8) | 0.202 |  | 589 (29.7) | 261 (37.2) | <0.001 |  | 411 (27.7) | 439 (36.7) | <0.001 |
| Disability (GARS) | 43 (35;51) | 26 (21;31) | <0.001 |  | 34 (27;44) | 24 (20;30) | <0.001 |  | 33 (26;43) | 28 (21;38) | <0.001 |  | 32 (25;41) | 31 (24;42) | 0.106 |
| Global cognitive function (MMSE) | 27 (24;28) | 28 (27;29) | <0.001 |  | 28 (26;29) | 28 (27;29) | <0.001 |  | 28 (26;29) | 28 (26;29) | <0.001 |  | 28 (26;29) | 28 (26;29) | 0.675 |
| Depressive symptoms (GDS-15) | 3 (1;5) | 1 (0;3) | <0.001 |  | 2 (1;4) | 1 (0;2) | <0.001 |  | 2 (1;4) | 0 (0;2) | <0.001 |  | 2 (1;5) | 1 (0;2) | <0.001 |
| Loneliness (Loneliness Scale of De Jong Gierveld) | 3 (1;5) | 2 (0;4) | <0.001 |  | 2(1;5) | 1(0;4) | <0.001 |  | 3 (1;5) | 1 (0;3) | <0.001 |  | 4 (2;6) | 1 (0;2) | <0.001 |
| Quality of Life (EQ-5D) | 0.65 (0.25;0.78) | 0.81 (0.68;0.89) | <0.001 |  | 0.69 (0.33;0.81) | 0.84 (0.77;1) | <0.001 |  | 0.69 (0.36;0.81) | 0.81 (0.72;1) | <0.001 |  | 0.72 (0.37;0.81) | 0.81 (0.68;0.89) | <0.001 |
| GP-contact time (minutes) | 160 (70-340) | 90 (40-150) | <0.001 |  | 120 (60-240) | 70 (30-130) | <0.001 |  | 110 (60-220) | 80 (40-170) | <0.001 |  | 110 (60-220) | 90 (40-180) | <0.001 |
| Data are numbers (%) or medians [IQR]  p-trend values were obtained with chi-square tests (categorical data) or linear regression analysis(continuous data)  GARS=Groningen Activities Restriction Scale, GDS-15=Geriatric Depression Scale, MMSE=Mini-Mental State Examination, EQ-5D=Euroqol-5D, GP=General practitioner  Individuals can have problems on more than one domain, therefore the number of participants adds up to a higher number than the study population  ‡ GP contact information was available for 1473 participants | | | | | | | | | | | | | | | |
