# Supplementary material for: Consequences of Interaction of Functional, Somatic, Mental and Social Problems in Community-Dwelling Older People
Source: PLoS One. 2015 Apr 21;10(4):e0121013. doi: 10.1371/journal.pone.0121013 (PMC4405543; doi:10.1371/journal.pone.0121013)
Supplement: S3 Table — *linear regression analyses modelled on the effect of the individual domains on the score on the health-indicator at after 12 months of follow up (adjusted for age, sex and baseline score of the health indicator). ¥ Loneliness Scale of De Jong Gierveld (DOC) [file pone.0121013.s005.doc]

| **S3 Table.** Association of the individual domains and the scores on health indicators at 12 months of follow-up (t12)^*^ | | | | | | | | | | | | |
| --- | --- | --- | --- | --- | --- | --- | --- | --- | --- | --- | --- | --- |
|  | GARS |  | MMSE |  | GDS-15 |  | Loneliness^¥^ |  | EQ-5D |  | GP-contact time (min) |  |
|  | β (95% CI) | p | β (95% CI) | p | β (95% CI) | p | β (95% CI) | p | β (95% CI) | p | β (95% CI) | p |
| Functional domain | 3.3 (2.5;4.0) | <0.001 | -0.6 (-0.8;-0.3) | <0.001 | 0.4 (0.2;0,6) | <0.001 | -0.08 (-0.3;0.1) | 0.42 | -0.11 (-0.13;-0.09) | <0.001 | 26 (-1.9;55) | 0.068 |
| Somatic domain | 1.0 (0.4;1.7) | 0.003 | -0.3 (-0.6;-0.06) | 0.016 | 0.5 (0.3;0.7) | <0.001 | 0.4 (0.2;0.6) | <0.001 | -0.09 (-0.12;-0.07) | <0.001 | 31 (5.7;55) | 0.016 |
| Mental domain | 1.1 (0.52;1.8) | <0.001 | -0.3 (-0.6;-0.01) | 0.039 | 0.6 (0.4;0.8) | <0.001 | 0.5 (0.3;0.7) | <0.001 | -0.09 (-0.12;0.07) | <0.001 | 34 (11;57) | 0.004 |
| Social domain | -0.3 (-0.6;0.5) | 0.930 | 0.01 (-0.2;0.3) | 0.962 | 0.3 (0.1;0.5) | <0.001 | 1.0 (0.8;1.1) | <0.001 | -0.03 (-0.05;-0.01) | 0.010 | 35 (15;56) | 0.001 |
| *linear regression analyses modelled on the effect of the individual domains on the score on the health-indicator at after 12 months of follow up (adjusted for age, sex and baseline score of the health indicator) | | | | | | | | | | | |  |
| ¥ Loneliness Scale of De Jong Gierveld | | | | | | | | | | | | |
